# Neural correlates of mindful emotion regulation in high and low ruminators

**DOI:** 10.1038/s41598-020-71952-5

**Published:** 2020-09-24

**Authors:** David Rosenbaum, Agnes M. Kroczek, Justin Hudak, Julian Rubel, Moritz J. Maier, Theresa Sorg, Lucca Weisbender, Lara Goldau, Douglas Mennin, David M. Fresco, Andreas J. Fallgatter, Ann-Christine Ehlis

**Affiliations:** 1 Department of Psychiatry and Psychotherapy, University Hospital of Tuebingen, Tübingen, Germany; 2 Center on Mindfulness and Integrative Health Intervention Development (C-MIIND), University of Utah, Salt Lake City, UT USA; 3 Psychotherapy Research Lab, Psychology and Sport Sciences, Justus-Liebig-University Giessen, Giessen, Germany; 4 Frauenhofer IAO | Center for Responsible Research and Innovation, Berlin, Germany; 5 Department of Psychological Sciences, University of Tuebingen, Tübingen, Germany; 6 Hunter College, City University of New York, New York, NY USA; 7 Department of Psychiatry, University of Michigan, Ann Arbor, MI USA; 8 Center of Integrative Neuroscience (CIN), Cluster of Excellence, University of Tuebingen, Tübingen, Germany; 9 LEAD Graduate School and Research Network, University of Tuebingen, Tübingen, Germany; 10 Institute for Social Research, University of Michigan, Ann Arbor, MI USA

**Keywords:** Human behaviour, Emotion, Social neuroscience

## Abstract

Depressive rumination is considered a prominent risk factor for the occurrence, severity, and duration of depressive episodes. A variety of treatment options have been developed to treat depressive rumination of which mindfulness based programs are especially promising. In the current study, we investigated the neural underpinnings of a short mindfulness intervention and mindful emotion regulation in high and low trait ruminators in an ecologically valid environment using functional near-infrared spectroscopy (fNIRS). Participants were randomly assigned to a mindfulness instruction (MT) group or an instructed thinking (IT) group. Participants in the MT group were trained to either focus their attention mindfully on their breath or their emotions, while the IT group focused their attention on the past or future. Afterwards, all participants underwent an emotion regulation paradigm in which they either watched negative or neutral movie clips. During both paradigms cortical hemodynamic changes were assessed by means of fNIRS. Participants in the MT group showed lower activity in the cognitive control network (CCN) during the focus on breath condition in comparison to the focus on emotion condition. Additionally, oxygenated hemoglobin in the MT group tended to be lower than in the IT group. Further, self-reports of emotional distress during the instruction paradigm were reduced in the MT group. During the emotion regulation paradigm, we observed reduced emotional reactivity in terms of emotional distress and avoidance in the MT group in comparison to the IT group. Furthermore, on a neural level, we observed higher CCN activity in the MT group in comparison to the IT group. We did not find any effect of rumination, neither on the intervention nor on the emotion regulation task. The results of this pilot study are discussed in light of the present literature on the neural correlates of mindfulness based interventions in rumination and emphasize the use of fNIRS to track neural changes in situ over the course of therapy.

## Introduction

Major Depressive Disorder (MDD) is considered a leading contributor to the global burden of disease^1^ and has a serious impact on social functioning and everyday routines. Despite the abundance of readily available treatment options, the prevalence and burden of MDD has not decreased, most probably due to moderate recovery rates and reduced stability of treatment effects^2^. Between one to two thirds of patients successfully treated with cognitive behavioral therapy (CBT) relapse within two years of initial treatment^3,4^. Relapse rates after discontinuation of pharmacotherapy are even higher^5–7^. One of the factors that increase the risk for relapse is habitual depressive rumination^8,9^, which is often defined as a pessimistic self-referential repetitive thinking style about problems with little or no goal- and change-orientation^10^. On a conceptual level, rumination lines up with and is correlated to other maladaptive perseverative phenomena such as worry and intrusive thought^8^. In addition to worsening relapse rates, depressive rumination is associated with further clinical outcomes, such as higher symptom severity, longer duration of depressive episodes and higher risk of suicide^8,11–15^. Further, rumination is associated with cognitive malfunctioning^16,17^, reduced emotional wellbeing, emotion dysregulation^18,19^, and higher physiological stress parameters^20^. On a neural level, rumination has been shown to be related to increased activity in areas of the cognitive control network (CCN) and default mode network (DMN) during induction paradigms^21–25^, decreased activity in cognitive tasks^26,27^ as well as aberrant functional connectivity between these networks^26,28,29^. The CCN is typically involved in tasks that require effortful control and top-down regulation and comprises areas such as the dorsolateral prefrontal cortex and posterior parietal cortex^30,31^. In contrast, the DMN involves areas such as the medial prefrontal cortex, posterior midline, precuneus and lateral temporal cortex, and is most active during rest^26,32,33^. With respect to emotion regulation, high trait ruminators show a pattern of decreased prefrontal activity during the downregulation of negative affect in prefrontal areas of cognitive control^27,34^ and increased activity in subcortical areas^34,35^.

In light of the increasing evidence indicating how depressive rumination complicates the severity and treatment efficacy of conditions such as MDD^36^, interventions for the acute treatment and relapse prevention have been developed that specifically target processes such as negative self-referentiality including depressive rumination^37–42^. With respect to acute treatment, interventions such as Emotion Regulation Therapy (ERT) incorporate techniques from CBT and mindfulness-based intervention to tackle pathological processes such as worrying and rumination. First empirical evidence suggests that ERT is indeed highly effective in reducing pathological self-referential thinking such as worry and rumination and symptoms in generalized anxiety disorder (GAD) and MDD^43–45^. Furthermore, a recent study by O’Toole et al.^46^ showed that ERT-related increase in cognitive reframing (CBT skill) and decentering (mindfulness skill) preceded decreased worry in GAD^46^. In terms of relapse prevention, a recent meta-analysis suggests that Mindfulness-based Cognitive Therapy (MBCT) decreases the risk for relapse by a hazard-ratio of 0.7 in comparison to active treatment^37^. In this meta-analysis, participants that received MBCT had an 11% reduced relapse risk as compared to subjects that did not receive MBCT (38% vs. 49%) within 60 weeks. Similarly, findings indicate that Rumination Focused Cognitive Behavioral Therapy as compared to usual care lowers the rate of relapses within a 6 months follow-up (9.5% vs. 53%)^38^.

As outlined above, mindfulness—the ability to direct one’s attention to the present moment and cultivate a non-judgmental awareness—is often a component of successful treatment of depressive rumination^47–50^. With respect to neural correlates, it is important to distinguish between studies of advanced meditators and novices as the latter usually activate more areas of the CCN during meditation^51,52^. Most likely, this difference is attributable to effects of acquiring and learning the skill of mindfulness in novices, e.g. needing more effortful control to achieve and maintain the meditative state. In general, mindfulness has been associated with activity in brain areas that are involved in emotion regulation (e.g., areas in the CCN and limbic regions), attentional control [e.g., anterior cingulate cortex (ACC)] and self-awareness (e.g., areas in the DMN). Meta-analytic data on different forms of meditation showed further overlapping activation in the limbic system, basal ganglia and medial prefrontal cortex for different forms of meditation^53^. In a study on the neural correlates of Mindfulness-Based Stress Reduction (MBSR) in GAD, Hölzel et al.^54^ showed that an 8-week MBSR program reduced amygdala activation and increased ventrolateral prefrontal activation during an affect labeling task. Further, frontolimbic connectivity increased in the treatment group and changes in activation and functional connectivity were associated with changes in clinical assessments^54^. These findings were replicated recently in a healthy cohort by Doll et al.^55^ who found that attention-to-breath mindfulness practice was associated with lateral and medial prefrontal activation and with fronto-limbic coupling during an emotion-regulation task in an fMRI investigation^55^. Aside from the reported long term intervention studies, first evidence from short-term laboratory manipulations suggests that mindfulness reduces induced negative mood^56,57^, as well as perceived stress during social evaluative situations^58^ and increases attentional control and immune reactivity^59^.

The current pilot study aimed to investigate the neural correlates of mindful emotion regulation in high and low trait ruminators. To this end, we employed the optical imaging method of functional near-infrared spectroscopy (fNIRS) during two paradigms in high and low trait ruminators. By using fNIRS we were able to assess cortical blood oxygenation in an ecologically valid environment, as fNIRS is able to be applied in various body positions (sitting, staying, walking) and is relatively subtle in terms of measurement related noises or hardware.

In the first phase of the study, 34 high trait ruminators and 33 low trait ruminators were randomized to either a mindfulness (MT) or instructive thinking (IT) group after a baseline assessment. In the mindfulness group, subjects were asked to cultivate either a mindfulness focus on the breath or mindfulness focus on experiences and emotions during 14 trials of 40 s duration. In the instructive thinking group, subjects were asked to direct their attention either to a situation in the past or in the future and think about this situation over and over again during 14 trials of 40 s duration. After each block, subjectively rated success, effort, emotional distress and attentional lapses were assessed. In the second study phase, all subjects completed an emotion regulation task during which subjects watched video clips with neutral and negative valence. After each trial, subjectively rated emotional distress and avoidance were assessed. Furthermore, emotion ratings, state rumination and state decentering were measured over the course of the experiment with questionnaires (baseline, after the short instruction paradigms, directly after the emotion regulation paradigm and 10 min following the completion of the emotion regulation paradigm).

Based on our prior work on rumination, we hypothesized that high ruminators would show generally elevated levels (main effect rumination) of state rumination, negative affect, emotional distress and avoidance, as well as higher reactivity following the emotion regulation task in comparison to baseline (interaction rumination*time). Further, as even brief mindfulness trainings have been shown to be effective on short-term emotion regulation, we expected that the mindfulness instruction would decrease the emotional reactivity in general (main effect) and within the high ruminators more strongly than in the low ruminators (ordinal interaction: instruction group*rumination*time).

With respect to cortical oxygenation (O_2_Hb) as measured with fNIRS, we expected that the MT group would show higher CCN activity than the control group, especially during mindful non-judgmental focus (interaction: intervention*condition). We investigated the effect of rumination in an exploratory manner during the instruction paradigm.

Additionally, we expected that the high ruminators would show reduced cortical oxygenation during the emotion regulation task in the CCN and that the trained instruction would normalize this reduction (interaction: instruction group*rumination*condition). For a detailed description of the hypotheses per paradigm and used post-hoc tests see Supplementary Table S1.

## Materials and methods

### Participants

Sixty-seven participants were recruited via email and flyers for this study. Exclusion criteria were acute physical illness, neurological disorders, substance abuse, chronic or acute diseases that affect brain functioning such as diabetes or kidney failure, cardiac arrhythmia as well as other cardiac diseases. The ethics committee of the University Hospital and University of Tuebingen approved this project and all subjects gave written informed consent. 34 high ruminators and 33 low ruminators were recruited for this study based on their response in the Ruminative Response Scale^60,61^. In total, 193 subjects completed the RRS online questionnaire. High ruminators were defined by an RRS mean score higher than 2.25 (percentile > 65), low ruminators by a score lower than 1.8 (percentile < 34). Cut-off criteria were defined as in previous studies in our group^62^, based on comparisons between depressed patients and non-clinical controls^26^ (see Supplemental Material Fig. S1).

Out of the 193 completers, 131 subjects that fulfilled our criteria were contacted. Out of those, 74 subjects were randomized. However, 7 subjects dropped out of the study during participation, in 6 cases before the measurement, and in 1 case during the measurement due to overly high emotional arousal during the emotion regulation task. Subjects were randomized to two different treatment groups: Mindfulness Training (MT) or Instructed Thinking (IT). Sociodemographic and clinical characteristics are shown in Table 1. In total, 10 subjects had some kind of experience with relaxation training (n = 2), autogenous training (n = 3) and/or meditation (n = 8). Eight of the 10 subjects just started with practicing within the last year, and 7 subjects reported to practice less than once per week or not at all on a regular basis. As no subject reported to practice mindfulness meditation for a substantial amount of time on a regular basis, we did not exclude subjects due to mindfulness practice. The treatment groups did not differ significantly in sex ratio. However, the low ruminators in the MT group showed a significantly higher mean age than the other three groups (all t-values > 2.17, *p* < 0.05, d > 0.7). As expected, low ruminators showed reduced levels in depressive symptomatology (BDI: F(1,62) = 22.161, *p* < 0.001, partial η^2^ = 0.15), habitual rumination (RRS: F(1,62) = 303.709, *p* < 0.001, partial η^2^ = 0.83), state rumination (state-rumination: F(1,62) = 18.120, *p* < 0.001, partial η^2^ = 0.23), negative affect (PANAS-NA: F(1,62) = 7.777, *p* < 0.01, partial η^2^ = 0.11) and increased levels of baseline measured habitual mindful acting (KIMS-act: F(1,62) = 8.552, *p* < 0.01, partial η^2^ = 0.12), acceptance (KIMS-acceptance: F(1,62) = 38.202, *p* < 0.001, partial η^2^ = 0.37) and decentering (EQ(1,62) ) = 5.292, *p* < 0.05, partial η^2^ = 0.08) (see Supplementary Table S3). As the study groups differed significantly in age, we repeated the reported analysis using age, sex and experience with relaxation/mediation as covariates within MANCOVAs (see Supplemental Material Table S5 and results).

**Table 1:** Demographic and clinical variables of the experimental groups at baseline.

Variable	MT group				IT group			
	Low ruminators		High ruminators		Low ruminators		High ruminators	
	Mean	(SD)	Mean	(SD)	Mean	(SD)	Mean	(SD)
Age (years)	28.6	(9.59)	23.2	(3.15)	22.9	(3.46)	22.3	(3.46)
Sex ratio (f/m)	12/4		15/3		12/5		9/7	
Prior knowledge (yes/no)	4/10		4/14		1/16		1/15	
BDI	4.4	(4.0)	9.8	(7.7)	3.3	(4.7)	11.0	(5.3)
RRS	1.5	(.21)	2.6	(.32)	1.6	(.17)	2.5	(.23)
EQ-trait	2.4	(.32)	2.3	(.57)	2.5	(.39)	2.1	(.32)
Baseline NA	1.1	(.14)	1.3	(.35)	1.2	(.20)	1.3	(.30)
Baseline SR	1.4	(.33)	1.9	(.64)	1.4	(.31)	1.9	(.75)
KIMS—observing	3.2	(.66)	3.7	(.55)	3.4	(.50)	3.5	(.50)
KIMS—describing	3.6	(.41)	3.7	(.60)	3.5	(.36)	3.3	(.55)
KIMS—acting	3.3	(.50)	2.9	(.48)	3.3	(.33)	2.9	(.75)
KIMS—acceptance	4.1	(.50)	3.3	(.63)	4.0	(.52)	3.0	(.62)

Prior knowledge = Number of subjects with experiences in meditation or relaxation training, RRS = Rumination Response Scale, EQ = Experiences Questionnaire, Baseline NA = Negative emotions as rated by the Positive and Negative Affect Schedule, Baseline SR = Baseline state rumination, KIMS = Kentucky Inventory of Mindfulness Skills.

### Procedures

**Figure 1:**
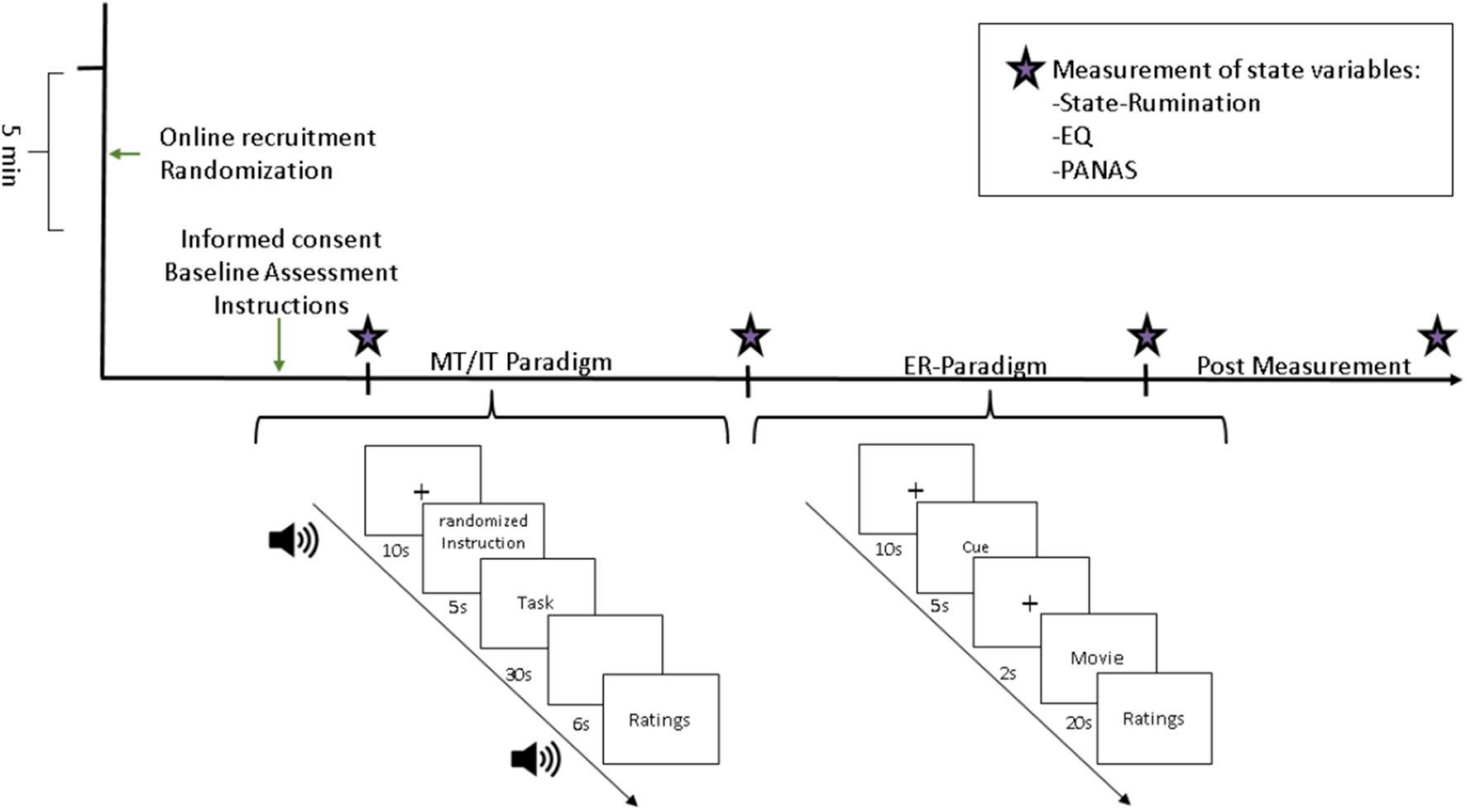
Experimental workflow and used paradigms. *MT* mindfulness instruction, *IT* instructed thinking, *ER* emotion regulation.

On the day of the measurement, participants completed baseline questionnaires while the fNIRS measurement was prepared (see Fig. 1). The baseline questionnaires consisted of sociodemographic data, the Beck Depression Inventory (BDI)^63^, Kentucky Inventory of Mindfulness Skills (KIMS)^64^, Experiences Questionnaire (EQ)^65,66^ in the trait form, the state form of the Positive and Negative Affect Schedule (PANAS)^67^ and a state rumination questionnaire^26,27^. Afterwards, subjects were given written information about the first task of the study: either the MT or IT. After reading the instruction (see supplemental material), subjects were asked to summarize their task and were instructed again verbally by the examiner. The MT group was instructed to either focus on their breath in the present moment while being non-judgmental (condition one: focus) or focus on their current feelings and emotions in a non-judgmental way while being in the present moment (condition two: equanimity). The two conditions were presented in randomized order and used to implement the two core elements of mindfulness: (1) focusing the attention in the present moment and (2) being non-judgmental about the present experience^68^. Furthermore, both of these aspects are present during mindfulness practice. While the main task during mindfulness meditation is, e.g., being focused on the breath non-judgmentally, this focus often becomes disturbed by emotionally loaded thoughts. In this case, meditators are instructed to recognize that they are not mindful anymore, and redirect their attention back to the breath while being non-judgmental about their feelings and thoughts.

The IT group was instructed to direct their attention towards a past (condition one: past) or future event (condition two: future) and to think about this event over and over again. The IT was designed as a control paradigm to the MT group, as thinking about the future is a non-mindful task (at least in non-meditators). Furthermore, we sought to create a control condition for the MT aside from baseline comparisons.

Both of the short interventions were similar in their design. First, subjects were asked if they had any questions about the task. Afterwards, subjects were instructed via audio to close their eyes. A 10 s baseline measurement followed before a peep-tone marked the start of the trial. During the first 10 s of the trial, subjects were instructed about their focus during this trial. Afterwards, subjects performed the task for 30 s. A second peep-tone marked the end of the task, followed by a 6 s inter-trial interval before subjects were instructed to open their eyes again. At the end of each trial, subjects gave ratings on perceived success, effort, emotional distress and attentional shifts during the trial. Subjects performed 14 blocks with randomized ordering of conditions. At the end of the completion of the short intervention, the MT group was instructed to stay in the mindful focus during the following emotion regulation task, while the IT group was left without instruction. We chose not to give specific instructions to the IT group to lower the risk for artificial neural activity (e.g. while subjects are actively trying to not regulate). Therefore, the emotion regulation task measures the mindful emotion regulation in the MT group and the emotional reactance in the IT group.

After this first phase of the study, subjects completed state measurements of decentering, negative emotion ratings and state-rumination. In the second study phase, all subjects participated in an emotion regulation task. During the paradigm subjects were asked to watch 28 different movie clips of 20 s length each with strongly negative or neutral valence. During the experiment, movie clips were presented in blocks, each consisting of 7 trials of a given valence. Movie clips were presented in an ABAB and BABA version in randomized order of movie blocks within each version. This procedure was used as some movie clips were in relation to each other, e.g. a suicide scene was cut to be used in 4 clips starting from the beginning of the suicide to the finding of the corpse by the parents of the suicide victim. Each trial consisted of a 10 s baseline followed by a 5 s prompt, instructing which trial would follow (anticipation phase). Next, a 2 s fixation cross was presented before the 20 s video clip started (watching phase). At the end of each clip, subjects rated their emotional distress and their avoidance during the movie clip on a 1 (not at all) to 9 (very much) Likert-scale. Avoidance was defined as the urge or act of fleeing the situation and withdrawing from the emotional experience. Negative clips included holocaust scenes (movie “The pianist”), suicide (“13 Reasons why”), murder (“American History X”), crying girl (“The fault is in our stars”), a dead baby (“Trainspotting”); neutral clips included informational film clips about sunflowers, gardening, chess, paper plane folding and knitting (for descriptive statistics per video see Supplementary Table S2).

After the emotion regulation task, subjects completed the state questionnaires two times: directly and 10 min after the task.

### fNIRS

Cortical activation was measured with a continuous wave, multichannel NIRS system (ETG-4000 Optical Topography System; Hitachi Medical Co., Japan) with a temporal resolution of 10 Hz. The same probeset placement was used as in previous studies of our group on the CCN in rumination^27,62,69^. It consisted of two frontal and one parietal probeset positioned on an Easycap with sponge rings for additional fixation.

After the assessment, data was further analyzed using MATLAB R2019a (MathWorks Inc, Natick, USA). We used the relatively new motion correction method of Fishburn et al.^70^: Temporal Derivative Distribution Repair (TDDR). Briefly, the method uses an adaptive algorithm to identify and correct sharp high amplitude movement artefacts based on the signal’s amplitude distribution^70^. Afterwards, we performed the correlation based signal improvement method of Cui et al.^71,72^ to reduce low amplitude artefacts that would not be captured by the TDDR. Further, interpolation of single artifact-loaded channels was performed, before data were bandpass filtered (0.1–0.01 Hz). As hemodynamic data might be confounded by global physiological artefacts through respiration, we performed a global signal reduction with a spatial Gaussian kernel filter^73^ with a standard deviation of σ = 40. Finally, the signal was z-standardized before event-related averages were computed.

In the MT and IT instruction paradigm, a 10 s baseline correction in a 70 s window was used. Mean activity was extracted between 10 and 40 s, as auditory instructions were given in the first 10 s of each trial. For data in the emotion regulation task, a 10 s baseline correction in a 32 s window was used for linear detrending. Data were averaged for the anticipation of negative and neutral videos (0–7 s) and the watching of negative and neutral videos (7–27 s).

Data was averaged in 5 regions of interest (ROI) comprising the superior parietal lobule (SPL), bilateral dlPFC and inferior frontal gyrus (IFG). ROIs were selected according to previous studies on the CCN^27,62,69^.

### Data analysis

Statistical analysis has been conducted with IBM SPSS State Statistics 24. Figures have been created with the ggplot2 package in R. Brain maps have been created with self-written MATLAB routines. Multiple comparisons of post-hoc tests were corrected by the Benjamini–Hochberg procedure. As this study was a pilot study to investigate the usefulness of a blocked mindfulness short intervention and the potential effects on emotion regulation, we conducted a sensitivity analysis given an alpha level of α = 0.05 and a power of β = 0.80 with G-power. Given the sample size of N = 67, we were able to detect effect sizes up to f^2^(V) = 0.19 for comparisons between two groups (main effect instruction group/rumination) with 4 dependent variables and f^2^(V) = 0.21 for 5 dependent variables, which are both moderate effect sizes. A within-between interaction needed to have an effect of f(V) = 0.34 for two groups (e.g. instruction group *condition) and f(V) = 0.42 for 4 groups (instruction group *rumination), which are moderate to large effect sizes.

We separately analyzed the first (instruction group) and second (emotion regulation) paradigm, as well as changes in emotion, decentering and state-rumination. In all analyses we used mixed MANOVAs with the between subject factors rumination (high vs. low) and instruction group (MT vs. IT) and the within-subject factor condition (MT: focus on breath vs. emotion; IT: focus on past vs. present; ER task: negative vs. neutral videos) of the related instruction. Dependent variables (DVs) were the behavioral scores (ratings) during the paradigm and NIRS data of the five ROI. Prior to the analysis, the assumptions of MANOVA were checked. Data for the behavioral ratings were transformed to achieve normal distribution. Post-hoc analysis was conducted on the DVs that further showed significant results in the univariate statistics. Univariate analyses following the MANOVAs were corrected by the Benjamini–Hochberg procedure. According to our hypothesis, we were mainly interested in main effects and interactions of the between subject factors. In each instruction group, we analyzed subjective ratings first as part of the manipulation check and subsequently fNIRS data.

#### Statistical analyses of paradigm 1: instruction groups

Behavioral data in the instruction groups was analyzed using a 2 (rumination: high vs. low) by 2 (instruction: MT vs. IT) by 2 (condition: focus/past vs. equanimity/future) MANOVA with 4 DVs (rating: attentional shifts vs. effort vs. emotional distress vs. success). As post hoc tests, we performed planned comparisons between (1) the groups and (2) the conditions within the groups (in MT: focus vs. equanimity; in IT: past vs. future). Interactions with the factor rumination were explored by comparing the subgroups (MT-rumination low, MT-rumination high, IT-rumination low, IT-rumination high) and the higher order interactions in the same manner (see Supplementary Table S1).

fNIRS data in the first paradigm was analyzed using a 2 (rumination: high vs. low) by 2 (instruction group: MT vs. IT) by 2 (condition: focus/past vs. equanimity/future) MANOVA with 5 DVs (ROI: left DLPFC, left IFG, right DLPFC, right IFG, SPL). Planned comparisons were equivalent to those of the behavioral data. Additionally, for descriptive purposes we performed tests of O_2_Hb values against baseline (t-test against zero) to assess the general activation pattern during the intervention paradigm (see Supplementary Analysis Table S4).

#### Statistical analyses of paradigm 2: emotion regulation task

In the emotion regulation task we directly analyzed difference scores of the experimental contrast (negative videos vs. neutral videos) for reasons of simplicity. Behavioral data in the emotion regulation task was analyzed using a 2 (rumination: high vs. low) by 2 (instruction group: MT vs. IT) MANOVA with 2 DVs (rating: emotional distress vs. avoidance). Post-hoc tests were performed in DVs with significant univariate effects and included comparisons between the instruction groups and subgroups of rumination (high ruminators MT vs. IT; low ruminators MT vs. IT) in the ratings.

fNIRS data were analyzed with a 2 (rumination: high vs. low) by 2 (instruction group: MT vs. IT) by 2 (phase: anticipation vs. watching) MANOVA for the contrast of the conditions (negative—neutral) on 5 DVs (ROI: left DLPFC vs. left IFG vs. right DLPFC vs. right IFG vs. SPL). Post-hoc tests included comparisons between the instruction groups and rumination groups separately for the different ROI (see Supplementary Table S1).

#### Statistical analysis of state variables over the course of the experiment

To analyze changes in negative emotion ratings, state-rumination and decentering, mixed MANOVAs with the factors instruction, rumination and time (measurement point) were conducted. We used time-related contrasts (linear, quadratic and cubic) to track changes over the course of the experiment. Planned comparisons included interactions of the time factor with instruction and rumination (see Supplementary Table S1).

### Ethics approval

The ethics committee at the University Hospital and University of Tuebingen approved this project and all subjects gave written informed consent. All used methods and procedures in this study were in accordance to the current guidelines of the World Medical Association's Declaration of Helsinki.

## Results

### First paradigm: MT vs. IT

#### Behavioral data

During the instruction, we observed a main effect of instruction group (F(4,60) = 5.618, *p* < 0.001, Wilk’s Λ = 0.728, partial η^2^ = 0.27), a main effect of condition (F(4,60) = 5.849, *p* < 0.001, Wilk’s Λ = 0.719, partial η^2^ = 0.28) and an interaction of condition by instruction group (F(4,60) = 6.652, *p* < 0.001, Wilk’s Λ = 0.693, partial η^2^ = 0.31). No main effect of or interaction with rumination was found (all *p* > 0.1).

**Figure 2:**
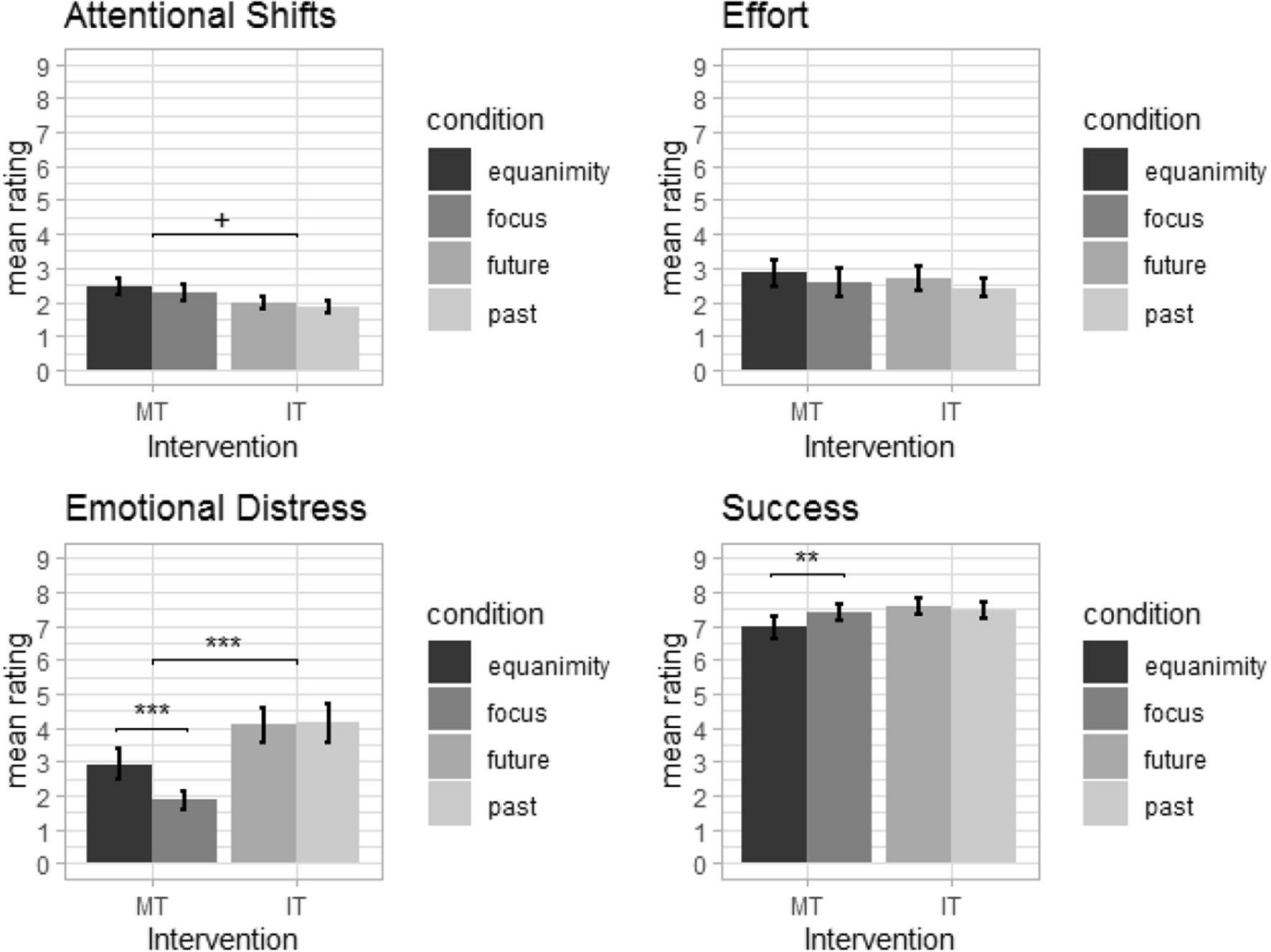
Ratings (Likert scale from 1 to 9) during the instruction paradigm in the MT group and IT group. ***p < 0.001, **p < 0.01, *p < 0.05, ^+^*p* < 0.05 without correction for multiple comparisons.

Univariate analysis of the main effect of instruction group was significant for the DVs emotional distress (F(1,63) = 18.765, *p* < 0.001, partial η^2^ = 0.23) and attention shifts (F(1,63) = 5.048, *p* < 0.05, partial η^2^ = 0.07); however, the latter finding was not significant after correction for multiple comparisons in the univariate ANOVAs. The main effects indicated lower emotional distress and marginally more attention shifts in the MT group in comparison to the IT group (see Fig. 2).

Further, the interaction of condition by group showed significant results for the DVs emotional distress (F(1,63) = 16.001, *p* < 0.001, partial η^2^ = 0.20) and perceived success (F(1,63) = 9.669, *p* < 0.01, partial η^2^ = 0.13). Post-hoc tests were only performed between the conditions of each instruction group, as the main effect already showed significant differences between the groups. Post-hoc tests revealed that the MT group showed reduced emotional distress (t(33) = 3.940, *p* < 0.001, d = 0.73) and higher perceived success of task completion (t(33) = 3.422, *p* < 0.01, d = 0.39) during the focus condition in comparison to the equanimity condition, while no differences in these variables were observable between the conditions of the IT group (see Fig. 2).

### Cortical activation

The analysis of fNIRS data during the instruction paradigm showed a marginally significant effect of instruction group (F(5,58) = 2.025, *p* < 0.1, Wilk’s Λ = 0.851, partial η^2^ = 0.15), a significant main effect of condition (F(5,58) = 3.631, *p* < 0.01, Wilk’s Λ = 0.762, partial η^2^ = 0.24) and a marginally significant interaction of condition by instruction group (F(5,58) = 2.354, *p* < 0.1, Wilk’s Λ = 0.831, partial η^2^ = 0.17). The marginal main effect of instruction group was characterized by a tendency towards reduced CCN activity in the MT group in comparison to the IT group.

**Figure 3:**
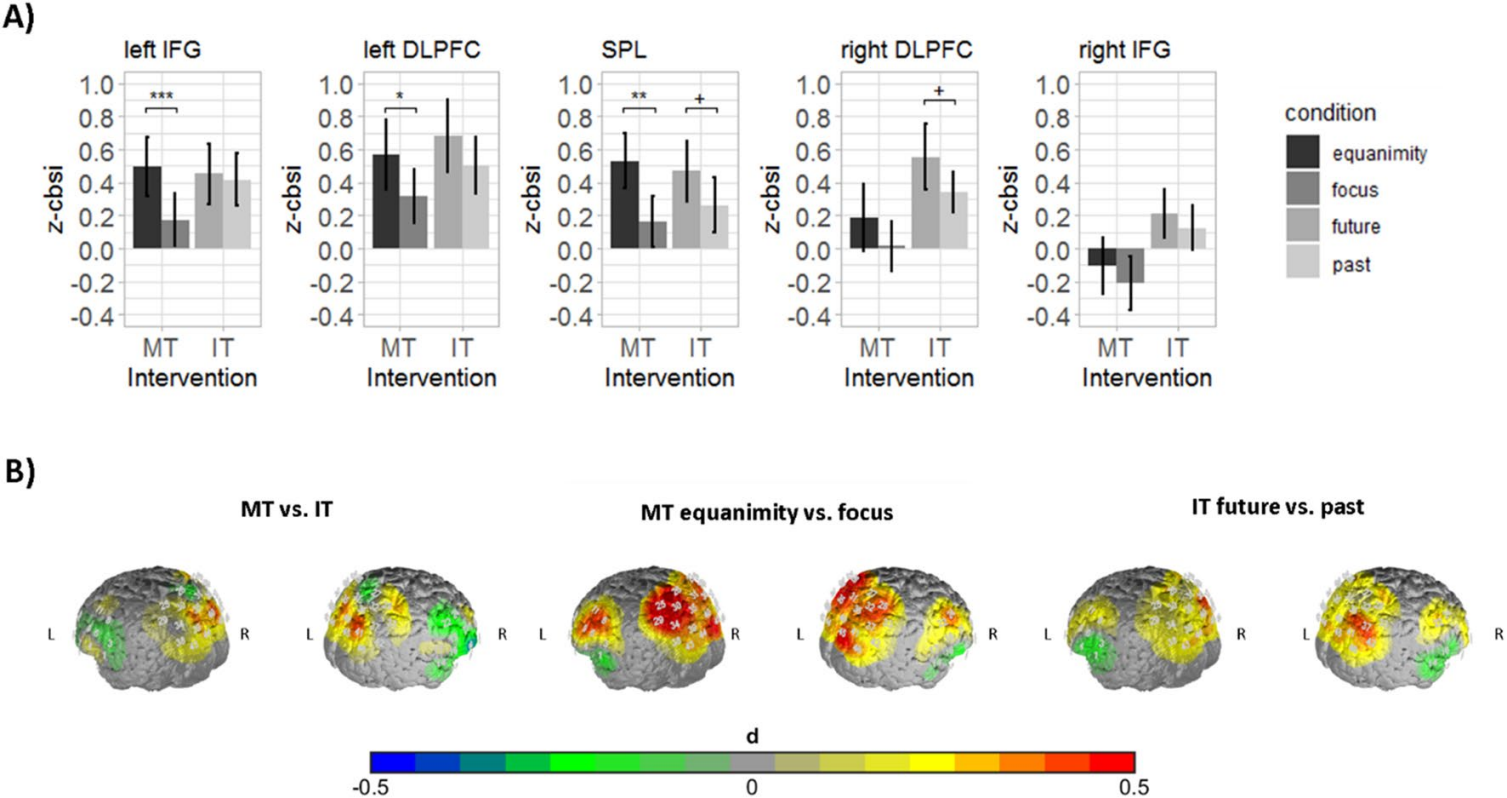
Activation during the short intervention. (**A**) Bar plots depicting the average z-score of the correlation based signal improvement (CBSI) O_2_Hb value for each region of interest (ROI) in the condition for the MT- and IT group. The focus condition in the MT group and the past condition in the IT group is colored in dark grey, the equanimity condition in the MT group and the future condition in the IT group is colored in light grey. *IFG* inferior frontal gyrus, *DLPFC* dorsolateral prefrontal cortex, *SPL* superior parietal lobule, ***p < 0.001, **p < 0.01, *p < 0.05, ^+^*p* < 0.05 without correction for multiple comparisons. (**B**) Contrast head maps for the observed effects. *MT* mindfulness instruction, *IT* instructed thinking, *L* left, *R* right. Brain maps were created using self-written MATLAB routines.

In summary, only the condition effect was significant, implying a similar pattern of activity in the equanimity/future condition and focus/past condition in the instruction groups. The univariate comparison of the main effect of condition showed that both groups combined had significantly higher activity in the equanimity/future condition than in the focus/past condition in the left IFG (F(1,62) = 8.295, *p* < 0.01, partial η^2^ = 0.12), left DLPFC (F(1,62) = 9.825, *p* < 0.01, partial η^2^ = 0.14), right DLPFC (F(1,62) = 8.121, *p* < 0.01, partial η^2^ = 0.12), and SPL (F(1,62) = 18.160, *p* < 0.001, partial η^2^ = 0.22). As combined comparisons of the instruction groups on the condition factor during the short intervention are somehow misleading, e.g. as the equanimity condition of the MT group is not directly comparable/equivalent to the future condition of the IT group, we reanalyzed the condition effect for both groups separately. Here again, we observed a main effect for condition in the MANOVA in both groups (MT: F(5,28) = 3.667, *p* < 0.05, Wilk’s Λ = 0.604, partial η^2^ = 0.40); IT: F(5,26) = 2.622, *p* < 0.05, Wilk’s Λ = 0.665, partial η^2^ = 0.33). Univariate analysis for the MT group indicated significantly higher activity in the equanimity than in the focus condition in left IFG (F(1,32) = 15.027, *p* < 0.001, partial η^2^ = 0.32), left DLPFC (F(1,32) = 7.154, *p* < 0.05, partial η^2^ = 0.18) and SPL (F(1,32) = 11.522, *p* < 0.01, partial η^2^ = 0.27). In contrast, no univariate comparison in the IT group survived the correction for multiple comparisons. Tendencies towards higher activity in the future in comparison to the past condition were observed in the left DLPFC (F(1,30) = 3.204, *p* < 0.1, partial η^2^ = 0.10), right DLPFC (F(1,30) = 5.013, *p* < 0.05, partial η^2^ = 0.14) and SPL (F(1,30) = 7.064, *p* < 0.05, partial η^2^ = 0.19). The different impact of the condition factor in both groups might further explain the marginally significant interaction of condition by instruction group in the main analysis (see Fig. 3).

### Second paradigm: emotion regulation task

#### Behavioral data

**Figure 4:**
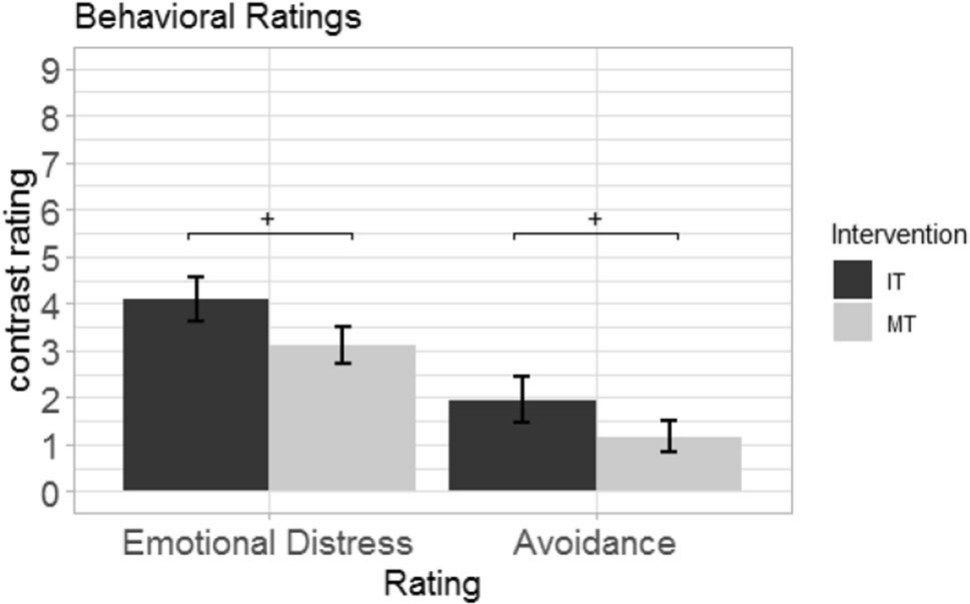
Mean ratings of emotional distress and avoidance during the emotion regulation task. ^+^*p* < 0.05 without correction for multiple comparisons.

After the short intervention, all subjects participated in the emotion regulation task. With respect to behavioral ratings during the emotion regulation task, we found a significant main effect of condition (negative vs. neutral) as indicated by a significant constant of the experimental contrast (F(2,60) = 135.483, *p* < 0.001, Wilk’s Λ = 0.181, partial η^2^ = 0.82). Univariate analysis indicated higher emotional distress (F(1,61) = 275.084, *p* < 0.001, partial η^2^ = 0.82) and higher avoidance (F(1,61) = 89.545, *p* < 0.001, partial η^2^ = 0.60) during watching of the negative movies in comparison to neutral movies (see Fig. 4). Further, we found a main effect of instruction group (F(1,62) = 3.160, *p* < 0.05, Wilk’s Λ = 0.905, partial η^2^ = 0.10). Univariate analysis showed significant results for emotional distress (F(1,61) = 5.099, *p* < 0.05, partial η^2^ = 0.08) and avoidance (F(1,61) = 4.781, *p* < 0.05, partial η^2^ = 0.07), but both effects did marginally not survive the correction for multiple comparisons (p_krit_ = 0.025, p_emp_ = 0.028).

No main effect of or interaction with rumination was found (all *p* > 0.1).

#### Cortical activation

With respect to cortical O_2_Hb levels during the emotion regulation task, we observed a main effect of the constant of the contrast values (negative-neutral movies) (F(5,57) = 12.855, *p* < 0.001, Wilk’s Λ = 0.470, partial η^2^ = 0.53), a main effect of instruction group (F(5,57) = 2.986, *p* < 0.05, Wilk’s Λ = 0.792, partial η^2^ = 0.21) and a main effect of phase (F(5,57) = 3.318, *p* < 0.05, Wilk’s Λ = 0.775, partial η^2^ = 0.23).

Univariate analysis of the constant term showed significantly increased O_2_Hb levels in the left IFG (F(1,61) = 19.705, *p* < 0.001, partial η^2^ = 0.24), left DLPFC (F(1,61) = 16.125, *p* < 0.001, partial η^2^ = 0.21), right DLPFC (F(1,61) = 7.599, *p* < 0.01, partial η^2^ = 0.11) and SPL (F(1,61) = 43.790, *p* < 0.001, partial η^2^ = 0.42), indicating increased cortical blood oxygenation in these brain areas during negative in comparison to neutral movie clips.

**Figure 5:**
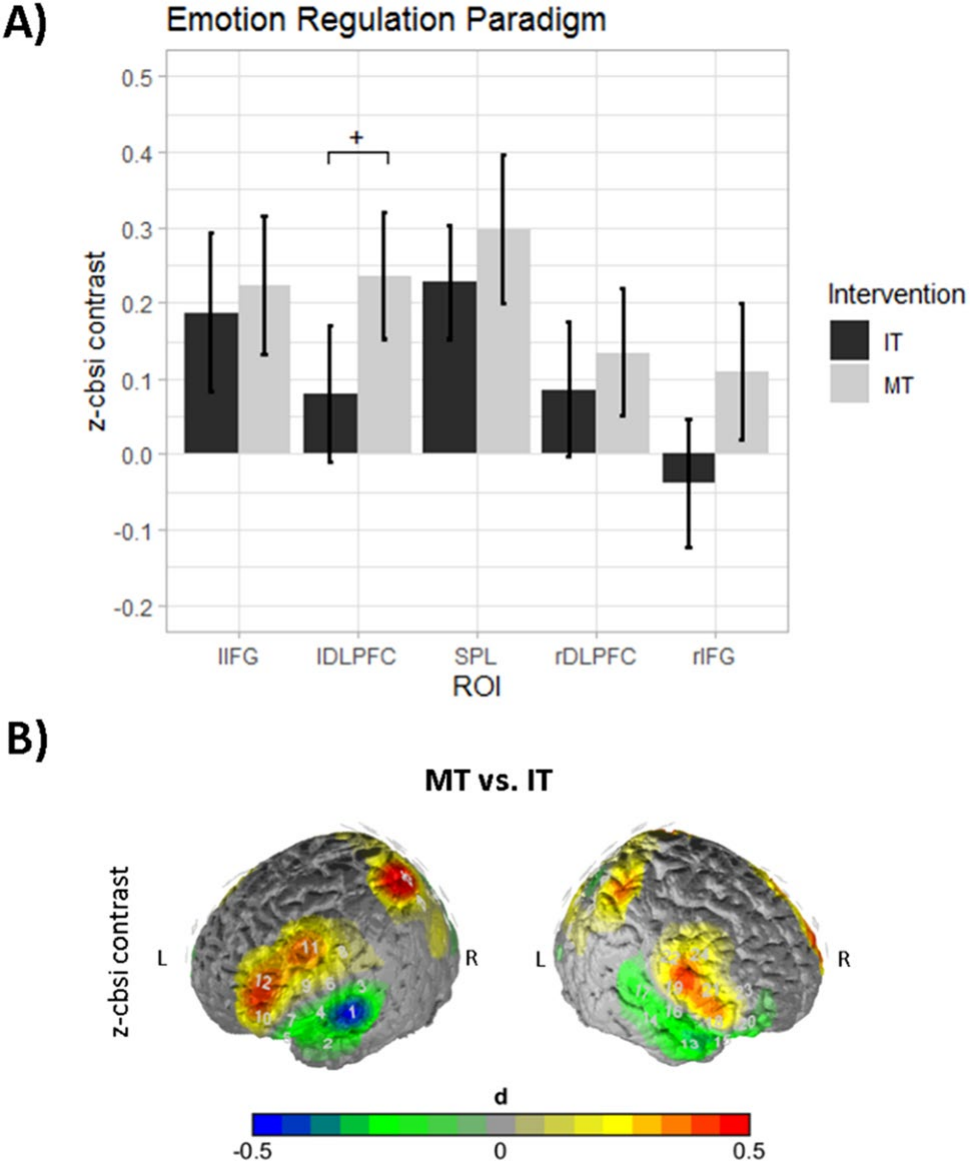
Activation during the emotion regulation task. (**A**) Bar plots depicting the main effect of instruction group. Dependent variable is the z-score of the correlation based signal improvement (CBSI) O_2_Hb value. ^+^*p* < 0.05 without correction for multiple comparison. (**B**) Contrast head maps for the observed effects. The contrast of the MT vs. IT group is depicted with values in effect size d. Warm colors indicate higher activity in the mindfulness group. Brain maps were created using self-written MATLAB routines.

The main effect of instruction group showed significantly increased O_2_Hb levels in the MT in comparison to the IT group in the left DLPFC (F(1,61) = 4.589, *p* < 0.05, partial η^2^ = 0.07), although the effect did not survive the correction for multiple comparisons for the univariate analysis, and a marginally significant effect in the right IFG (F(1,61) = 3.394, *p* < 0.1, partial η^2^ = 0.53) (see Fig. 5).

Finally, the univariate analysis of the main effect of phase showed significantly increased O_2_Hb levels during anticipation in comparison to watching in the left IFG (F(1,61) = 12.793, *p* < 0.001, partial η^2^ = 0.17) and SPL (F(1,61) = 4.586, *p* < 0.05, partial η^2^ = 0.07).

No main effect of or interaction with rumination was found (all *p* > 0.1).

### Changes in decentering, subjective emotion, and state-rumination over the course of the experiment

**Figure 6:**
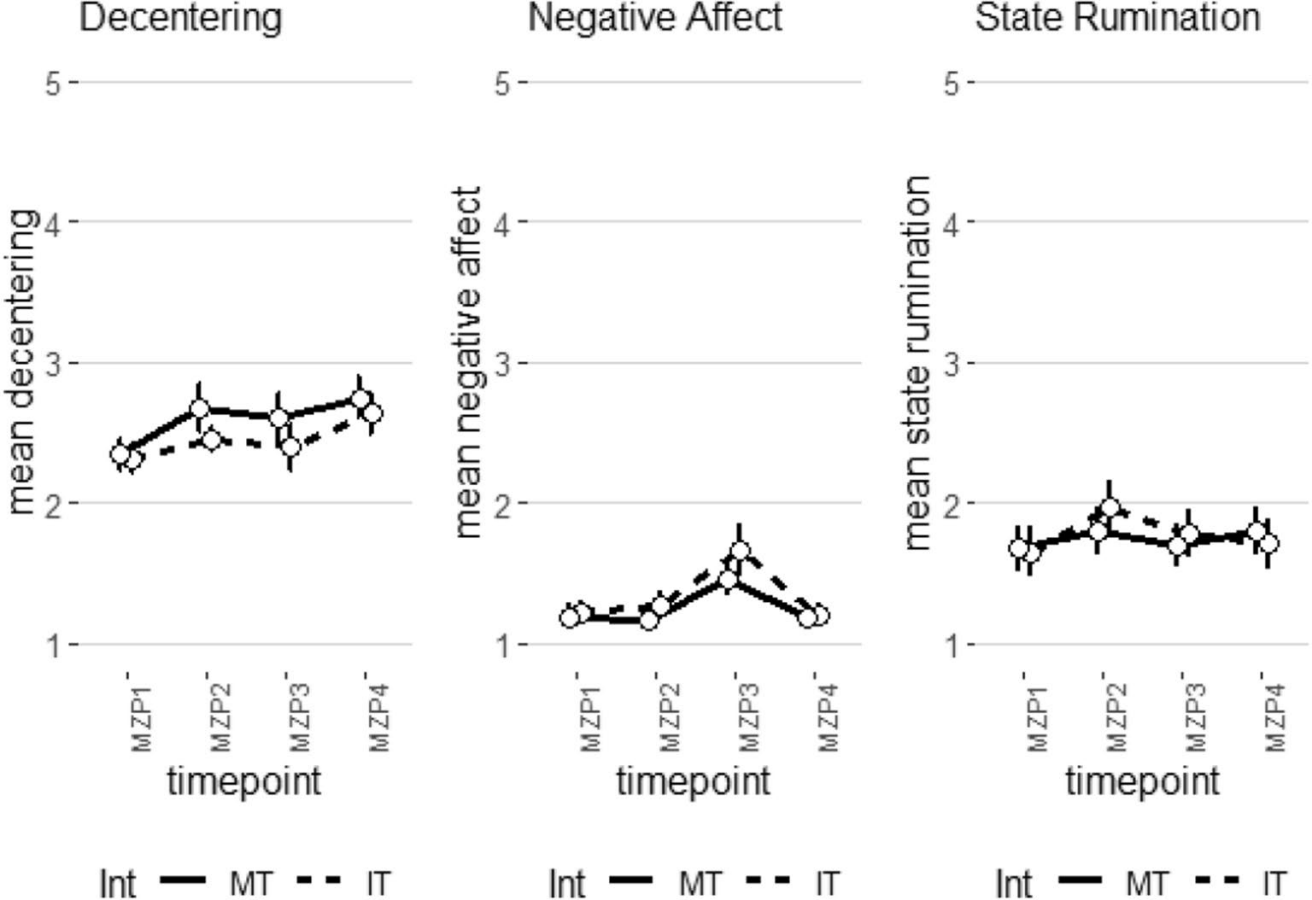
Changes in decentering, negative affect and state rumination over the course of the experiment in the instruction groups. MZP1 = measurement before the short intervention, MZP2 = measurement after the short intervention/before the emotion regulation task, MZP3 = measurement directly after the emotion regulation task, MZP4 = measurement 10 min after the emotion regulation task.

Lastly, we analyzed changes in decentering, negative emotion and state rumination over the course of the experiment. Our results of the multivariate analysis of variance showed a significant main effect for rumination (F(3,57) = 6.454, *p* < 0.001, Wilk’s Λ = 0.746, partial η^2^ = 0.24) and time (F(9,51) = 13.182, *p* < 0.001, Wilk’s Λ = 0.301, partial η^2^ = 0.70). The univariate analysis of the main effect of rumination showed significantly increased state rumination (F(1,59) = 19.157, *p* < 0.001, partial η^2^ = 0.25), and negative affect (F(1,59) = 4.187, *p* < 0.05, partial η^2^ = 0.07) in the high ruminators. However, the latter effect was not significant after correction for multiple comparisons. The main effect of time showed significant effects in all DVs: negative affect (F(3,177) = 25.939, *p* < 0.001, partial η^2^ = 0.31), state rumination (F(3,177) = 5.633, *p* < 0.001, partial η^2^ = 0.09) and decentering (F(3,177) = 8.911, *p* < 0.001, partial η^2^ = 0.13). The change in negative affect was characterized by a quadratic (F(1,59) = 29.686, *p* < 0.001, partial η^2^ = 0.34) and cubic (F(1,59) = 42.293, *p* < 0.001, partial η^2^ = 0.42) change with a relatively steady level until the beginning of the emotion regulation task, an increase in negative affect after this task and a decrease 10 min later (see Fig. 6). For decentering the linear cubic (F(1,59) = 17.157, *p* < 0.001, partial η^2^ = 0.22) and cubic (F(1,59) = 6.727, *p* < 0.05, partial η^2^ = 0.10) term reached significance. In this case, the effects were driven by a linear increase over the course of the experiment with a first peak after the short intervention, a subsequent decrease after the emotion regulation task and a maximal peak 10 min following the end of the emotion regulation task (see Fig. 6). Finally, with respect to state rumination, post-hoc testing indicated a quadratic (F(1,59) = 6.569, *p* < 0.05, partial η^2^ = 0.10) and cubic (F(1,59) = 11.071, *p* < 0.01, partial η^2^ = 0.16) change. The effect was driven by an increase in state rumination following the short intervention, a decrease after the emotion regulation task and a steady level 10 min later (see Fig. 6).

### Reanalysis controlling for age, sex and experience with relaxation or meditation

Although we randomized subjects to the instruction groups, there were significant differences in the average age between the treatment conditions. Further, sex was not equally distributed within the sample and prior experience to any kind of relaxation or meditation showed tendencies to an unequal distribution as well. Therefore, we repeated the analysis with these variables as covariates with MANCOVA. In Supplementary Table S5, we summarize the effects of the prior analysis and the controlled analysis. In the reanalysis, all previously reported results were still significant, but the main effect of instruction group on the behavioral ratings during the emotion regulation task showed only marginally significant results. When inspecting the univariate analysis of the effect, the effect of the intervention on avoidance was no longer significant, but the effect on emotional distress showed significance. Therefore, the effect of the instruction group on behavioral ratings during the emotion regulation task has to be interpreted with caution. However, the change in significance level might also be attributed to the limited power of this pilot sample.

## Discussion

The present study aimed to investigate an adapted mindfulness short intervention and corresponding effects on behavioral and neural parameters of emotion regulation in low and high habitual ruminators. To this end, 67 habitual high and low ruminators either underwent a mindfulness or instructed thinking short intervention while functional near-infrared spectroscopy (fNIRS) was assessed. Afterwards, all subjects watched either affectively neutral or negative movie clips during an emotion regulation task.

As expected, we observed reduced emotional distress in the MT in comparison to the IT group on a behavioral level. Further, emotional distress within the MT group was lower in the focus when compared to the equanimity condition. Likewise, perceived success was reduced in the equanimity in comparison to the focus condition. No differences in these variables were observed between the conditions of the IT group. On a neural level, contrary to our hypothesis, we observed a marginally significant effect of the instruction group towards reduced activity in the CCN in the MT group. Further, we found a significant condition effect in the whole sample, reflecting higher activity in the equanimity/future condition than the focus/past condition in the instruction groups. As both groups did perform different tasks, we subsequently performed the post-hoc tests for the condition effect separately for both groups, without comparing the instruction groups directly (within-subject comparison vs. between-subject comparison). Post-hoc analysis showed an expected higher activity during the equanimity than the focus condition in the MT group in the left DLPFC, left IFG and SPL. The supplementary analysis of the activation pattern in the two conditions further indicated that this effect was due to no CCN activation during the focus condition but during the equanimity condition. In the IT group differences between conditions were not significant after correction for multiple comparisons, pointing only to tendencies towards higher activation in the future than in the past condition in the bilateral DLPFC and SPL. However, the supplementary analysis of the activation during the two conditions showed that the past as well as the future focus induced activity in the CCN different from zero. In the design of the study and the formulation of the hypotheses, we did not expect that the IT conditions would induce such high CCN activity. The differences between the MT conditions, however, point towards two different neural processes that are characterized by different levels of emotional distress and perceived success in the task completion. One explanation for this might be that the mindful focus on the emotional state provoked effortful control for emotion regulation to maintain a distanced mindful focus on the emotional state. This interpretation is in line with the model of model-based and model-free emotion regulation of Etkin et al.^74^. In their framework, the authors suggest that—during model-based emotion regulation—plans and internal models are used to guide behavior and that areas of the CCN are involved in this process^74^. The mindful focus on the emotional state versus the breath might just be more related to follow internal plans, which would be associated with higher model-based control. Interestingly, this interpretation might also be related to findings from meditation stages. For example, Hasenkamp et al.^75^ postulate that during meditation four stages are involved: mindwandering, awareness of mindwandering, attention shifting and sustained attention. In their study, Hasenkamp and colleagues observed increased activity within areas of the default mode network during mindwandering, increased activity within the salience network during awareness of mindwandering and increased activity in the cognitive control network during attention shifting and sustained attention^75^. Associations of CCN activation and attentional control/shifting have also been shown in other fNIRS and fMRI studies^52,69,76–79^. Correspondingly, we propose that our results might reflect a naturally occurring effect that is present during mindfulness meditation when reflecting on emotions in a mindful state. The non-judgmental reflection of these emotional processes may be accompanied by increased activity in areas of the cognitive control network, before attention is reallocated, similar to the observed increased O_2_Hb levels during the equanimity condition.

Following the short intervention, all subjects underwent an emotion regulation task during which emotional video clips were first cued and then watched by all participants. Interestingly, on a behavioral level we again observed a main effect of the instruction group on behavioral ratings of emotional distress and avoidance, although both effects were significant only without correction for multiple comparisons in the univariate analysis. Therefore, this effect needs to be interpreted with caution, but hints towards a marginally significant improvement when looking at both variables separately. However, emotional distress and emotional avoidance were moderately positively correlated in this study (r = 0.60, *p* > 0.001) and in light of the significant multivariate test might be interpreted in terms of improved overall affective well-being. This effect is well in line with the work of Arch and Craske^57^ who found that a short mindfulness induction reduces negative affect and increases willingness to be confronted with negative material in comparison to an unfocussed attention and worry intervention^57^. In line with this, Creswell et al.^58^ observed reduced perceived stress and increased cortisol responses during the Trier Social Stress Test in subjects after 3 days of a mindfulness training in comparison to a control training. Additionally, the training was most effective in subjects that showed low pre-existing levels in dispositional mindfulness^58^. From this point of view, we suggest that the training of a mindful state within the utilized block design is comparable to the effects of mindfulness on mood found in the literature^56,58, 59^.

On a neural level we observed a main effect of the constant indicating higher CCN activity during negative vs. neutral movie clips and a main effect of phase that was characterized by increased cortical oxygenation already in the anticipation of the negative movie clips. Similar effects have been reported with other emotionally relevant material^80,81,^ which underlines the assumption that the regulation of the emotional process already starts with the anticipation of emotional events. Further, in the multivariate analysis we observed a main effect of instruction group implying increased CCN activity within the MT group in the experimental contrast negative versus neutral videos. However, the univariate analysis showed that this effect was mainly driven by the left DLPFC and was only significant without correction for multiple comparisons. Therefore, this effect has to be interpreted with caution and can only be related to the overall CCN activation. However, the finding of improved affective well-being and increased CCN activity after a mindfulness instruction is in line with the existing literature on the topic. A prior study on the effects of trait mindfulness on CCN activity during emotion regulation implicated a positive association of mindfulness and activity in the CCN during reappraisal^82^. Similarly, trait mindfulness was positively associated with prefrontal activity during affect labeling in an fMRI study^83^. Further evidence comes from an fMRI study of Lutz et al. (2014), who used a short mindfulness intervention before an emotion regulation task was assessed. They observed increased prefrontal activation during expectation of negative material in the mindfulness group relative to controls^84^. In another study, Hölzel et al.^54^ observed increased prefrontal activity in an affect labeling task following an MBSR intervention in GAD^54^. Nonetheless, our results must be interpreted with caution, as the univariate analysis for the behavioral and neural effects was only significant without correction for multiple comparisons. However, as this study was designed as a pilot study, we might suspect that increases in the dose of intervention might increase the observed tendencies found in this study. Further, we hypothesize that the overall effect of the blocked instruction paradigm, which differs substantially from traditional meditation settings in terms of timing and interruptions, might have the potential to be effective in a higher dosage.

Importantly, the factor of habitual rumination showed no significant effect, neither in the instruction paradigm nor in the emotion regulation task. In previous investigations using clinical analogue samples, we found encouraging effects in high habitual ruminators, despite the non-clinical groupings^27,62^. In general, it must be noted that reactivity in negative emotion and state rumination were rather low. As expected, high trait ruminators showed elevated general levels of state rumination and negative emotion. Surprisingly, interactions of instruction group, rumination and time were not significant, indicating that time-related changes in state rumination, negative affect and decentering were not dependent on these factors. The first explanation for this might be that the implemented factors to influence these dependent variables—e.g. the intervention and the instructed thinking task—were not strong enough to have an impact. Interestingly, the significant main effect of time indicates that state rumination increased in all subjects regardless of the instruction group, implying also increases in state rumination in the MT group following the instruction paradigm. It might be the case that the MT instruction had indeed some kind of negative side effects. Other studies have found that mindfulness based interventions may have “side effects” in terms of actualization and realization of emotionally loaded problems during meditation^85^. This effect may be in accordance with higher emotional distress in the MT group while focusing on one’s emotional state with a mindful focus. Another explanation may be that the instruction was too short for influencing ruminative tendencies.

Furthermore, state rumination did not increase during the emotion regulation task in high ruminators, which emphasizes the use of subjectively relevant emotional situations for the induction of state rumination^27,62^. Indeed, in subjectively relevant stress paradigms like those used by our group, the reactive negative emotion and state rumination were much higher than in the current study. It may be the case that group differences were not significant in this study, as the absolute effect of the paradigms utilized on subjective emotion and state rumination was rather small. In future studies on the effects of mindful emotion regulation, it might be of advantage to use paradigms such as the Trier Social Stress Test^86,87^.

Despite these promising findings, some limitations have to be noted. First, this study was conceptualized as a pilot study to investigate the implementation of a block-designed mindfulness instruction and the estimation of potential effect. Although the results are encouraging, the power of the used sample size is limited, resulting in the conflicting results following the correction for multiple comparisons. In future studies the estimated effect sizes of the short intervention in this study might be used to a priori estimate a sufficient sample size for the correction of multiple comparisons. Further, fNIRS only allows measurement of the upper layers of the cortex, because the penetration depth of the NIR light is limited^88,89^. However, in terms of ecological validity, fNIRS is the best available option for measuring cortical hemodynamic changes^90^. Further, it is important to bear in mind that the current study used a non-clinical population and future studies are needed to support the usage of the implemented mindfulness training in clinical samples. However, because one aim of the current study was to investigate the effects of the paradigm, our sample used also had some advantages. For example, high ruminators were medication-free, meaning our study was free from confounding of the observed effects with medication status of the high ruminating group. Also, although the sample was non-clinical, high ruminators showed significantly elevated levels of subclinical depression scores, indicating that this subgroup is potentially at-risk^90–93^. Another limitation concerns the instruction during the emotion regulation paradigm. During the emotion regulation paradigm, we did not give direct instructions how to regulate in the IT group, as we wanted to contrast a naturally occurring spontaneous emotion regulation in the IT group versus mindful emotion regulation in the MT group. However, without a direct instruction how to regulate, the IT group might have used a variety of different emotion regulation strategies. On the other hand, control instructions—such as not to regulate—themselves might induce neural activity, which is why we decided to design the paradigm in the current way. In future investigations it might also be interesting to investigate the spontaneous emotion regulation strategies in the non-intervention group in detail. Lastly, although most probably due to the limited sample size of this pilot study, the investigated subgroups of this study showed significant differences with respect to their age. However, controlling for the age of participants did not affect the results of our analysis. This is most probably due to the relatively low differences between groups in age, as all subject groups were on average between 22 and 29 years old. Most age-related changes in brain functioning occur in certain developmental windows. Following adolescence, brain functioning may be comparable across different age groups (e.g. 22 and 29 year old subjects) within adults until age-related decline starts. A last limitation concerns the study design. The development of control conditions for laboratory mindfulness inductions is rather complicated. We did not choose to use a non-intervention group—e.g. just resting for 15 min—as subjects in such a group could be mindful while performing the control condition. Instead, we used a non-mindful instructed thinking instruction that might have contributed to the differences between the groups. In future studies, it may be preferable to investigate a third non-intervention group. However, such study designs are cost and time expensive. In future studies it should be assessed if subjects practice Yoga more explicitly, as such a practice could improve mindfulness skills. Furthermore, in future studies additional physiological measures (e.g. heart rate) might be assessed, and larger sample sizes should be collected to allow the analysis of brain-behavior-correlations.

In conclusion, we showed that an fNIRS mindfulness block design showed promising results on brain activation and behavioral indices of emotion regulation. Moreover, different mindfulness states were accompanied by different involvement of areas of the cognitive control network demonstrated in a within-subject comparison. The results of this study show that the intervention paradigm might be useful in future studies to investigate the neural correlates of mindfulness-based interventions in situ using fNIRS. In future studies, the current training paradigm should be further investigated in clinical populations and longitudinal investigations to track changes in CCN activity through longer training periods.

## Supplementary information

Supplementary Information.
