# Supplementary material for: Neural correlates of mindful emotion regulation in high and low ruminators
Source: Sci Rep. 2020 Sep 24;10:15617. doi: 10.1038/s41598-020-71952-5 (PMC7518445; doi:10.1038/s41598-020-71952-5)
Supplement: Supplementary file 1 — Supplementary Information. [file 41598_2020_71952_MOESM1_ESM.docx]

**Supplemental Material: Neural correlates of mindful emotion regulation in high and low ruminators**

David Rosenbaum^1^, Agnes M. Kroczek^1^, Justin Hudak², Julian Rubel³, Moritz J. Maier^4^, Theresa Sorg^5^, Lucca Weisbender^5^, Lara Goldau^5^, Douglas Mennin^6^, David M. Fresco^7^, Andreas J. Fallgatter^1,8,9^, Ann-Christine Ehlis^1,9^

^1^ Department of Psychiatry and Psychotherapy, University Hospital of Tuebingen, Tuebingen, Germany

^2^ Center on Mindfulness & Integrative Health Intervention Development (C-MIIND), University of Utah, Salt Lake City, UT, USA

^3^ Psychotherapy Research Lab, Psychology and Sport Sciences, Justus-Liebig-University Giessen, Giessen, Germany

^4^ Frauenhofer IAO | Center for Responsible Research and Innovation, Berlin, Germany

^5^ Department of Psychological Sciences, University of Tuebingen, Germany

^6^ Hunter College, City University of New York, New York, NY, USA

^7^ Department of Psychology, Kent University, Kent, OH, USA

^8^ Center of Integrative Neuroscience (CIN), Cluster of Excellence, University of Tuebingen, Germany

^9^ LEAD Graduate School and Research Network, University of Tuebingen, Tuebingen, Germany

**Corresponding Author:**

David Rosenbaum

Calwerstraße 14

72076 Tübingen

Germany

email: david.rosenbaum@med.uni-tuebingen.de

Phone: 00497071 29-83609

**Keywords: Mindfulness, Emotion regulation, Rumination, functional Near-infrared Spectroscopy (fNIRS), Cognitive Control Network, neural correlates**


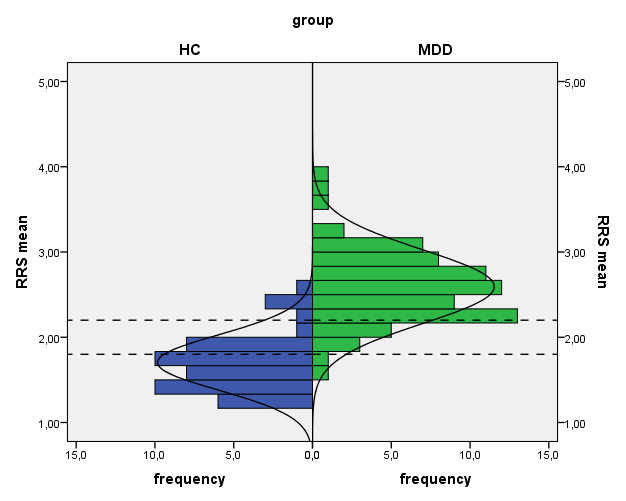


**Supplementary Figure S1:** Data from published and unpublished RRS scales from patients with MDD (green; n=75) and healthy controls (blue; n=45). Diagnosis and status as healthy control was assured by structured clinical interviews. Dotted lines indicate cut-off values for the low ruminators and high ruminators in this study.

**Supplemental Material: instruction for the mindfulness group**

The following experiment is about mindfully dealing with yourself and your emotions. The experiment is divided into two parts:

First, you will perform a mindfulness task to cultivate a mindful attentional state. Second, you will watch emotionally-charged videos. During the viewing, you will be asked to handle your thoughts and emotions in a calm, distanced and non-judgemental way.

Being mindful consists of two aspects that we will practice in the following trials: awareness in the present moment and non-judgement.

1. Intentionally focusing your attention on the present moment; being in the here and now. To accomplish this, you have to be fully focused and present in the current moment. To focus your attention on the present moment, you have to concentrate on your own breath. Please focus on your perceptions while breathing. Notice your sensations from time to time without categorizing them into good or bad. You may find that your mind wanders. That’s okay. Just notice that this is happening and redirect your attention back to your breath.
2. Non-judgement. This aspect of mindfulness is about handling your perceptions, emotions and thoughts gently. Please try to notice what you are feeling at the moment, like a distant observer. Pay attention to your emotions regardless of whether they are “pleasant” or “unpleasant”. You may find yourself judging or avoiding emotions (like “I don´t want that”). That’s okay. Just notice that this is happening and redirect your attention back to a calm, distanced and non-judgemental observer-perspective.

This aspect of mindfulness may be the hardest part of this exercise. Maybe it will help you to visualize yourself as an old and steadfast tree. Over many decades you observed calmly what happened around you. In this way, you are now able to better notice your perceptions. Obviously, you will categorize them as “pleasant” or “unpleasant” automatically. Being mindful means to not avoid or suppress them. Notice your emotions as they are and try to accept them. In case you observe yourself as not calm, that’s okay, don´t set yourself under pressure. Notice this tension, try not to judge it, and redirect your attention back to your mindful focus. This is the compassionate aspect of mindfulness: In case you register self-critical thoughts, ruminations or worries, notice them and redirect your attention, calm and non-judgmental, to your emotional perceptions in the here and now. If these emotions are “too strong”, you may try to view them from greater distance. Nevertheless, your focus should lie on existent emotions and perception and not on thinking processes, because thought often leads us away from the present moment, so that we aren´t in the here and now anymore.

Again, to summarize: Mindfulness is bringing attention to the present moment and perceiving everything as a distant, non-judgmental, calm and compassionate observer.

These two aspects of mindfulness will be practiced in different blocks. You will be instructed via speakers before each block. First, you will be asked to close your eyes during a 10 second baseline measurement. Try to sit as relaxed and still as possible. Afterwards, you will hear a tone, indicating imminent instruction as to which mindfulness exercise you will soon be practicing: “mindful focus on your breath” or “mindful focus on your emotions”. In both cases, all aspects of mindfulness should be realized: (1) To be in the here and now and (2) to perceive everything non-judgmentally; only the emphasis is different. In the condition “mindful focus on your breath”, try to practice primarily the aspect of being “in the here and now” and then to focus on non-judgmental perception. Let your breath flow freely and observe your perceptions while breathing. In the condition “mindful focus on your emotions”, prioritize focus on your emotions and perceptions in a calm and non-judgmental way, then focus on being in the here and now. In the end, you should perceive the here and now in a non-judgemental, calm, distant and self-compassionate way.

In the beginning, you will be asked to close your eyes. After the instruction as to which block will follow, you will practice for 30 seconds per trial. The end of every trial will be announced by a tone. Finish the exercise but keep your eyes CLOSED. After a couple of seconds, you will be asked to open your eyes again. It is fundamental for data acquisition with NIRS to only open your eyes when you are asked to. In the end, you will be presented with some questions about how well you implemented the instruction, how exhausting the trial was and how emotionally “distressed” you were. After answering the questions, the next trial will start. The exercise will last for about 15 minutes and spans 14 trials.

In the following section, the second part of the experiment with the videos will be conducted which will be instructed separately.

Do you have any questions? Please go and get the experimenter and explain again in your own word what mindfulness is and what you will have to do in the exercise.

**Supplemental Material: instruction for the instructed thinking group**

In the following experiment we aim to investigate how emotional films are perceived. The experiment is divided into two parts:

In the first part, you will focus your thoughts to the future and to the past. In the second part of the experiment, emotional videos will be shown to you.

The first part of the experiment will be as follows: You will be instructed via speakers before each block. First, you will be asked to close your eyes during a 7 second baseline measurement. Try to sit as relaxed and still as possible. Afterwards, you will hear a tone, indicating the imminent instructions for the next task: “focus on the future” or “focus on the past”. In both conditions, you should let your mind wander in the indicated direction. It is not important whether you think about things that have happened moments before, what will happen or how far your mind wanders in time. You may think about what you still have to do today or this week, or what you did before the experiment or last week. Please try to think about the same event each trial, or a sequential event (i.e. for the future task, an event further in the future; for the past task, an event further in the past).

After the block instructions, you will practice for 30 seconds per trial. The end of every training session will be announced by a tone. Finish the exercise, but keep your eyes CLOSED. After a couple of seconds, you will be asked to open your eyes again. It is fundamental for data acquisition with NIRS to only open your eyes when you are asked to. In the end, you will be presented with some questions about how well you implemented the instruction, how exhausting the trial was and how emotionally “distressed” you were. After answering the questions, the next trial will start. The exercise will last for about 15 minutes spanning 14 trials.

In the following section, the second part of the experiment with the videos will be conducted which will be instructed separately.

Do you have any questions? Please go and get the experimenter and explain again in your own words what your task is and what you will have to do in the exercise.

| **Paradigm** | **Independent Variable** | **Dependent Variable** | **Hypothesis** | **Post-Hoc Tests** |
| --- | --- | --- | --- | --- |
| First Paradigm: Intervention paradigm  (behavioral) | - Intervention (MT vs. IT) - Rumination (low vs. high) - Condition (focus/ past vs. equanimity/ future) | Ratings:   - Attention Shifts - Effort - Emotional distress - Success | 1. The MT group will show lower emotional distress than the IT group 2. Differences between & within conditions and higher-order interactions were explored in an undirected hypothesis as comparisons of the within-subject factors within each group | If significant, univariate analysis of DV:   1. MT vs. IT 2. MT equanimity vs. MT focus 3. IT past vs. IT future 4. Exploratory analysis of rumination |
| First Paradigm: Intervention paradigm  (fNIRS) | - Intervention (MT vs. IT) - Rumination (low vs. high) - Condition (focus/ past vs. equanimity/ future) | ROI:   - Left IFG - Left DLPFC - Right IFG - Right DLPFC - SPL | 1. The MT group will show higher CCN activity than the IT group during the intervention paradigm 2. Within the MT group, CCN activity will be higher in the equanimity condition than in the focus condition 3. Differences between future and past focus in the IT-group were explored with an undirected hypothesis 4. Differences between high- and low ruminators were explored with an undirected hypothesis | If significant, univariate analysis of DV:   1. MT vs. IT 2. MT equanimity vs. MT focus 3. IT past vs. IT future 4. Exploratory analysis of rumination |
| Second Paradigm: Emotion regulation paradigm (behavioral) | - Intervention (MT vs. IT) - Rumination (low vs. high) | Contrast of the conditions (negative-positive)  DVs:   - Emotional distress - Avoidance | 1. The high ruminators will show higher emotional distress than the low ruminators if they participated in the IT group 2. The MT group will show reduced emotional distress and avoidance in the experimental contrast | If significant, univariate analysis of DV:   1. MT vs. IT 2. High rumination IT vs. high-rumination MT 3. Low rumination MT vs low-rumination IT |
| Second Paradigm: Emotion regulation paradigm (fNIRS) | - Intervention (MT vs. IT) - Rumination (low vs. high) | Contrast of the conditions (negative-positive)  DVs:   - Left IFG - Left DLPFC - Right IFG - Right DLPFC - SPL | 1. Higher CCN activity during negative affective videos in comparison to neutral videos 2. Higher CCN activity in low ruminators than in high ruminators in the experimental contrast (negative–neutral) 3. Normalized CCN activity in high ruminators that participated in the MT-group | If significant, univariate analysis of DV:   1. Main effect of constant 2. High rumination vs. low rumination 3. High rumination MT vs. high rumination IT 4. Low rumination MT vs. low rumination IT |
| Changes in Mood, state rumination and decentering over the course of the experiment | - Intervention (MT vs. IT) - Rumination (low vs. high) - Time-point | DVs:   - PANAS - State-rumination questionnaire - EQ | 1. We analyzed changes in these variables in an undirected hypothesis to check for potential long-term effects of the intervention and baseline differences between the groups | 1. Linear, quadratic and higher-order time contrasts |

Supplementary Table S1. Used paradigms, independent and dependent variables, hypothesis and post-hoc tests. MT = Mindfulness Intervention, IT = Instructed Thinking, rum = Rumination, CCN = Cognitive Control Network, DLPFC = dorsolateral prefrontal cortex , IFG = inferior frontal gyrus, SPL = superior parietal lobule , PANAS = Positive and Negative Affect Schedule, EQ = Experiences Questionnaire

|  | mean | | SD | |  | mean | | SD | |
| --- | --- | --- | --- | --- | --- | --- | --- | --- | --- |
| neutral | R 1 | R 2 | R 1 | R 2 | negative | R 1 | R 2 | R 1 | R 2 |
| Video 1 | 1.4 | 1.2 | 0.8 | 0.9 | Video 1 | 4.2 | 2.1 | 1.8 | 1.6 |
| Video 2 | 1.2 | 1.3 | 0.5 | 1.1 | Video 2 | 4.9 | 3.3 | 2.0 | 2.4 |
| Video 3 | 1.3 | 1.5 | 0.8 | 1.4 | Video 3 | 6.7 | 5.4 | 1.8 | 2.4 |
| Video 4 | 1.3 | 1.4 | 0.7 | 1.3 | Video 4 | 5.9 | 4.2 | 2.0 | 2.4 |
| Video 5 | 1.3 | 1.2 | 0.7 | 0.8 | Video 5 | 4.8 | 2.9 | 2.1 | 2.2 |
| Video 6 | 1.2 | 1.2 | 0.4 | 0.5 | Video 6 | 5.1 | 3.0 | 2.3 | 2.4 |
| Video 7 | 1.2 | 1.3 | 0.6 | 0.6 | Video 7 | 5.6 | 3.2 | 2.4 | 2.4 |
| Video 8 | 1.1 | 1.4 | 0.4 | 0.9 | Video 8 | 4.7 | 3.0 | 1.9 | 2.0 |
| Video 9 | 1.1 | 1.3 | 0.2 | 0.9 | Video 9 | 5.9 | 4.4 | 2.4 | 2.8 |
| Video 10 | 1.1 | 1.1 | 0.4 | 0.2 | Video 10 | 4.3 | 2.8 | 2.1 | 2.0 |
| Video 11 | 1.3 | 1.1 | 0.6 | 0.3 | Video 11 | 4.7 | 1.9 | 1.8 | 1.2 |
| Video 12 | 1.2 | 1.0 | 0.8 | 0.0 | Video 12 | 4.2 | 1.8 | 1.9 | 1.3 |
| Video 13 | 1.3 | 1.2 | 0.6 | 0.8 | Video 13 | 4.1 | 1.9 | 2.4 | 1.8 |
| Video 14 | 1.2 | 1.3 | 0.5 | 0.8 | Video 14 | 4.3 | 1.8 | 2.3 | 1.5 |
| Video 15 | 1.7 | 1.4 | 1.1 | 1.0 | Video 15 | 4.5 | 3.1 | 2.8 | 2.5 |
| Video 16 | 1.4 | 1.3 | 0.8 | 0.8 | Video 16 | 6.2 | 4.5 | 2.5 | 3.0 |
| Video 17 | 1.5 | 1.2 | 1.1 | 0.7 | Video 17 | 5.4 | 3.5 | 2.6 | 2.8 |
| Video 18 | 1.3 | 1.2 | 0.8 | 0.6 | Video 18 | 4.4 | 2.6 | 2.6 | 2.2 |
| Video 19 | 1.2 | 1.2 | 0.6 | 0.6 | Video 19 | 4.8 | 2.2 | 2.6 | 1.7 |
| Video 20 | 1.5 | 1.3 | 0.8 | 1.0 | Video 20 | 5.3 | 2.3 | 2.6 | 1.9 |
| Video 21 | 1.2 | 1.2 | 0.4 | 0.5 | Video 21 | 5.8 | 3.9 | 2.4 | 2.9 |
| Video 22 | 1.5 | 1.1 | 1.5 | 0.5 | Video 22 | 4.2 | 2.7 | 2.2 | 2.1 |
| Video 23 | 1.4 | 1.1 | 1.1 | 0.4 | Video 23 | 4.9 | 2.3 | 2.1 | 1.8 |
| Video 24 | 1.2 | 1.4 | 0.6 | 1.3 | Video 24 | 5.3 | 3.4 | 2.6 | 2.5 |
| Video 25 | 1.2 | 1.2 | 0.6 | 0.4 | Video 25 | 4.5 | 1.6 | 2.7 | 0.9 |
| Video 26 | 1.3 | 1.2 | 0.6 | 0.4 | Video 26 | 4.5 | 1.6 | 2.4 | 0.8 |
| Video 27 | 1.2 | 1.2 | 0.6 | 0.4 | Video 27 | 5.2 | 3.1 | 2.4 | 2.5 |
| Video 28 | 1.4 | 1.1 | 0.7 | 0.4 | Video 28 | 4.8 | 2.7 | 2.4 | 2.3 |

**Supplementary Table S2.** Ratings per Video for neutral and negative Videos. R1 = Rating 1, emotional distress; R2 = Rating 2, avoidance; SD = Standard deviation. The Rating was on a Likert-scale from 1 (not at all) to 9 (very much)

|  | **High Ruminators** | | **Low Ruminators** | |  |  |
| --- | --- | --- | --- | --- | --- | --- |
|  | **mean** | **(SD)** | **mean** | **(SD)** | **d** |  |
| RRS | 2.57 | 0.28 | 1.53 | 0.19 | 4.28 | *** |
| BDI | 10.38 | 6.61 | 3.87 | 4.32 | 1.16 | *** |
| EQ-trait | 2.20 | 0.46 | 2.44 | 0.36 | -0.57 | * |
| Baseline NA | 1.28 | 0.31 | 1.11 | 0.17 | 0.67 | ** |
| Baseline SR | 1.93 | 0.67 | 1.36 | 0.31 | 1.08 | *** |
| KIMS – observing | 3.58 | 0.53 | 3.35 | 0.58 | 0.43 |  |
| KIMS – describing | 3.47 | 0.60 | 3.54 | 0.38 | -0.13 |  |
| KIMS – acting | 2.92 | 0.61 | 3.31 | 0.41 | -0.73 | ** |
| KIMS – acceptrance | 3.20 | 0.64 | 4.06 | 0.50 | -1.50 | *** |

**Supplementary Table S3.** Differences between high and low ruminators in clinical variables.

**Supplementary analysis: Differences from zero in the experimental conditions during the intervention paradigm.**

With respect to levels of cortical oxygenated blood during the intervention, we observed activation significantly different from zero mostly in the IT group conditions in the left IFG, left DLPFC, right DLPFC and SPL. In contrast, significantly increased activation in the MT group was predominantly found in the left IFG, left DLPFC and SPL during the equanimity condition, but only in the left DLPFC in the focus condition (see table S4).

|  | MT group | | | |
| --- | --- | --- | --- | --- |
|  | Focus | | Equanimity | |
| ROI | t-value | p-value | t-value | p-value |
| left IFG | 1.60 | 0.119 | **4.11** | **0.000** |
| left DLPFC | 2.86 | 0.007 | **3.88** | **0.000** |
| right IFG | -1.93 | 0.063 | -0.90 | 0.372 |
| right DLPFC | 0.12 | 0.903 | 1.36 | 0.183 |
| SPL | 1.52 | 0.138 | **4.62** | **0.000** |
|  |  |  |  |  |
|  | IT group | | | |
|  | Past | | Future | |
|  | t-value | p-value | t-value | p-value |
| left IFG | **3.78** | **0.001** | **3.51** | **0.001** |
| left DLPFC | **4.24** | **0.000** | **4.44** | **0.000** |
| right IFG | 1.31 | 0.199 | 2.02 | 0.052 |
| right DLPFC | **3.96** | **0.000** | **3.95** | **0.000** |
| SPL | 2.24 | 0.033 | **3.66** | **0.001** |

**Supplementary Table S4**. Results of the t-tests against baseline values (tests against zero). Note that only bold p-values are significant after correction for multiple comparisons. ROI = Region of interest, IFG = Inferior frontal gyrus, DLPFC = Dorsolateral prefrontal cortex, SPL = Superior parietal lobule

|  | MANOVA | MANCOVA correcting for age,sex and experience |
| --- | --- | --- |
| Paradigm 1: Behavioral | - main effect: intervention(F(4,60) = 5.618, p < .001, Wilk’s Λ = .728, partial η² = .27) - main effect: condition (F(4,60) = 5.849, p < .001, Wilk’s Λ = .719, partial η² = .28) - interaction: condition*intervention (F(4,60) = 6.652, p < .001, Wilk’s Λ = .693, partial η² = .31) | - main effect: intervention(F(4,57) =4.702, p < .01 Wilk’s Λ =.752 partial η² = .25) - main effect: condition (F(4,57) = 6.302, p < .001, Wilk’s Λ = .693, partial η² = .31) - interaction: condition*intervention (F(4,57) = 5.688, p < .001, Wilk’s Λ = .715, partial η² = .29) |
| Paradigm1: fNIRS | - main effect: condition (F(5,58) = 3.631, p < .01, Wilk’s Λ = .762, partial η² = .24) | - main effect: condition (F(5,55) = 3.517, p < .01, Wilk’s Λ = .758, partial η² = .24) |
| Paradigm2: Behavioral | - significant constant: (F(2,60)=135.483, p<.001, Wilk’s Λ = .181, partial η²=.82) - **main effect: intervention (F(1,62) = 3.160, p < .05, Wilk’s Λ = .905, partial η² = .10)** | - significant constant: (F(2,57)=132.936, p<.001, Wilk’s Λ = .177, partial η²=.82) - **main effect: intervention (F(1,57) = 3.160, p < .1, Wilk’s Λ = .918, partial η² = .08)** |
| Paradigm 2: fNIRS | - significant constant (F(5,57)=12.855, p<.001, Wilk’s Λ = .470, partial η² = .53) - main effect: intervention (F(5,57)=2.986, p<.05, Wilk’s Λ = .792, partial η² = .21) - main effect: phase (F(5,57)=3.318, p<.05, Wilk’s Λ = .775, partial η² = .23) | - significant constant (F(5,54)=14.413, p<.001, Wilk’s Λ = .428, partial η² = .57) - main effect: intervention (F(5,54)=2.993, p<.05, Wilk’s Λ = .782, partial η² = .22) - main effect: phase (F(5,54)=3.749, p<.01, Wilk’s Λ = .742, partial η² = .25) |
| State variables | - main effect: rumination (F(3,57)=6.454, p<.001, Wilk’s Λ = .746, partial η² = .24) - main effect: time (F(3,57)=13.182, p<.001, Wilk’s Λ = .301, partial η² = .70) | - main effect: rumination (F(3,54)=5.813, p<.01, Wilk’s Λ = .756, partial η² = .24) - main effect: time (F(9487)=13.981, p<.001, Wilk’s Λ = .276, partial η² = .72) |

**Table S5. Supplementary analysis:** Results of MANCOVA controlling for the variables age, sex and experience with relaxation/meditation
